# Adherence to the screening program for HBV infection in pregnant women delivering in Greece

**DOI:** 10.1186/1471-2334-6-84

**Published:** 2006-05-09

**Authors:** Vassiliki Papaevangelou, Christos Hadjichristodoulou, Dimitrios Cassimos, Maria Theodoridou

**Affiliations:** 1 2^nd ^Department of Pediatrics, University of Athens, Children's' Hospital "A. Kyriakou", Goudi 11527, Athens, Greece; 2 Department of Hygiene and Epidemiology, University of Thessaly, Larissa 41222, Greece; 3 Department of Pediatrics, University of Thrace, Alexandroupoli 68100, Greece; 4 1^st ^Department of Pediatrics, University of Athens, Children's' Hospital "A. Sophia", Goudi 11527, Athens, Greece

## Abstract

**Background:**

Hepatitis B infection (HBV) is a major Public Health Problem.

Perinatal transmission can be prevented with the identification of HBsAg(+) women and administration of immunoprophylaxis to their newborns. A national prevention programme for HBV with universal screening of pregnant women and vaccination of infants is in effect since 1998 in Greece.

**Methods:**

To evaluate adherence to the national guidelines, all women delivering in Greece between 17–30/03/03 were included in the study. Trained health professionals completed a questionnaire on demographic data, prenatal or perinatal screening for HBsAg and the implementation of appropriate immunoprophylaxis.

**Results:**

During the study period 3,760 women delivered. Prenatal screening for HBsAg was documented in 91.3%. Greek women were more likely to have had prenatal testing. HBsAg prevalence was 2.89% (95%CI 2.3–3.4%). Higher prevalence of HBV-infection was noted in immigrant women, especially those born in Albania (9.8%). Other risk factors associated with maternal HBsAg (+) included young maternal age and absence of prenatal testing. No prenatal or perinatal HBsAg testing was performed in 3.2% women. Delivering in public hospital and illiteracy were identifiable risk factors for never being tested. All newborns of identified HBsAg (+) mothers received appropriate immunoprophylaxis.

**Conclusion:**

The prevalence of HBsAg in Greek pregnant women is low and comparable to other European countries. However, immigrant women composing almost 20% of our childbearing population, have significant higher prevalence rates. There are still women who never get tested. Universal vaccination against HBV at birth and reinforcement of perinatal testing of all women not prenatally tested should be discussed with Public Health Authorities.

## Background

Hepatitis B infection (HBV) is a major Public Health Problem. It is estimated that more than 350 million people are chronic HBV carriers worldwide. About one forth of them will develop chronic hepatitis and cirrhosis and could develop hepatocellular carcinoma eventually [1]. The probability of becoming a chronic carrier is inversely related to age at the time of infection [2,3]. Although only 5–10% of adults infected with HBV will become chronic carriers, neonatal infection almost always leads to a chronic carrier state (90%) whereas 30–60% of children infected during the first five years of life will become chronic HBV carriers [4]. The precise mechanism of this has not yet been defined but it has been attributed to the immaturity of the immune system of newborns and young children and their inability to mount an effective CTL response against HBV infection[5,6]. Acute and chronic HBV infections are usually asymptomatic during childhood. Up to 25% of infants and older children who acquire chronic HBV infection will eventually develop HBV-related hepatocellular carcinoma or cirrhosis [7].

The risk of perinatal transmission of HBV is 70% – 90% for infants born to mothers who are HBsAg and HBeAg positive, but it decreases to 5% – 20% for infants born to anti-HBe positive mothers [2]. Children of HBV-infected mothers remain at high risk of acquiring HBV infection by person-to-person (horizontal) transmission during the first 5 years of life [8].

More than 90% of the perinatal HBV infections can be prevented with the identification of HBsAg-positive pregnant women so that their newborns can receive hepatitis B vaccine and hepatitis B immune globulin (HBIG) soon after birth [9,10]. If a woman delivering has an unknown HBsAg status, the first dose of hepatitis B vaccine should be administered to the newborn as soon as possible. If this mother is found HBsAg(+) or her status remains unknown, HBIG should be administered within the first week of life[2]. Worldwide, it has also been recommended that hepatitis B vaccine should be integrated into routine vaccination schedules for infants, usually as a part of the World Health Organization's Expanded Programme on Immunization, especially in populations in which HBV infection is acquired during childhood [11,12].

In Greece, selective vaccination of high-risk groups was initiated in 1982. A national prevention programme for hepatitis B with universal screening of pregnant women and universal vaccination of infants and adolescents is in effect since 1.1.98. It is recommended that all infants are vaccinated for hepatitis B, however, birth dose of hepatitis B vaccine is administered only to newborns of HBsAg-positive mothers.

According to recent seroepidemiologic studies among army recruits [13] and blood donors[14] the prevalence of HBV carriers is below 1% making therefore Greece a country of low endimicity. On the other hand however, the influx of refugees from countries with higher endimicity (Eastern Europe and other Balkan countries, mainly Albania) pose the threat of increasing incidence of chronic HBV infection among people residing in Greece. In a recent prospective study in a large Maternity Hospital in Athens, the prevalence of HBsAg positivity was 2,8%. Only 63.1% of women had been tested during prenatal care. Moreover, risk factors associated with HBV carrier state included residence in a rural area, being an immigrant or young < 25 y.o) [15].

The aim of this study is to assess whether guidelines for the prevention of perinatal HBV infection are followed properly in Greece in both national and regional level five years after their implementation.

## Methods

### Surveillance of the adherence to the guidelines for the prevention of perinatal HBV infection

All pregnant women delivering in Greece between 17/03/03 and 31/03/03 were included in this observational study. All obstetric clinics of the country in public, private and University Hospitals were included in the study. After getting permission from the ethics committee and Public Health Department of the Ministry of Health we contacted by mail all Clinic Directors. Each clinic appointed one contact person for the study. In most instances, the head midwife responsible for the delivery room was the contact person for the study. She was responsible for completing an anonymous questionnaire for every woman delivering, which included demographic data (age, origin, educational level and address) as well as data on prenatal care and more specifically on prenatal screening for HBsAg (if she had documented screening during prenatal care, when, where and whether the result was known). Furthermore, if prenatal screening for HBsAg was not documented, we noted whether examination of the HBsAg serostatus (serology testing by ELISA) of the pregnant woman upon admission to the delivery room was ordered and whether appropriate immunoprophylaxis (intramuscular administration of the first dose of hepatitis B vaccine and 0,5 ml HBIG simulataneously but at a different site within 12–24 hours of life) was given to the newborn. To reinforce compliance to the study, frequent telephone calls to all contact persons were made and visits to different regions of Greece were performed during the two weeks of the study.

### Statistical analysis

A database was created in EPI-Info2002 (Epidemiological software of the Center for Diseases Control) and all data recruited from the questionnaires were transferred to that database in order to be analyzed. Data analysis was performed using EPI-Info 2002 and SPSS. Total prevalence of HBsAg(+) was calculated by adding women diagnosed during prenatal screening and those diagnosed for the first time in the delivery room (perinatally). Specific prevalence was calculated in each group of mothers. Chi-square test was used to compare qualitative values, whereas for quantitative values Students' t-test or non-parametric Mann-Whitney/Wilcoxon test were used whenever appropriate.

## Results

Most obstetric clinics agreed to participate in this study (137/138). During the study period (17–30/03/03) 3,760 questionnaires were completed.

Most women were of Greek origin (79.97%), 10.88% were born in Albania, 2.28% were of gipsy origin whereas 6.86% of women were immigrants from different countries. Women were almost equally distributed between public (46.7%) and private (53.3%) clinics. Interestingly, 1677 (44.6%) of women from all over Greece, delivered in Athens.

The prevalence of HBsAg(+) women delivering in Greece during the study period was 2.89% (95%CI 2.3–3.4%) of all tested. Women of Greek origin had significant lower prevalence of HBsAg(+) 1.7% (95%CI 1.3–2.3%) compare to Albanian women 9.8%, (95%CI 7.1–13.3%), immigrants from other countries 5.7% (95%CI 2.9–9.0%) and women of gipsy origin 3.6% (95%CI 0.8–10.2) Table (1).

**Table 1:** Study population, prevalence of HBsAg(+) and prenatal screening for HBsAg by origin.

ORIGIN	No (%)	Prevalence of HBsAg(+)**	Prenatal screening for HBsAg
GREEK	3007 (79.97%)	46/2705(1.7%)***	2467/2641(93.4%)***
ALBANIAN	409 (10.88%)	36/368(9.8%)	227/288 (78.8%)
IMMIGRANT*	258 (6.86%)	13/228(5.7%)	104/119 (87.4%)
GIPSY	86 (2.28%)	3/83(3.6%)	31/43 (72.1%)
TOTAL	3760	98/3384(2.89%)	2856/3128 (91.3%)

* other than Albanian

** Total prevalence in women tested either prenatally or perinatally

*** Statistically significant difference among Greek women versus non-Greek in HBsAg (+) prevalence and in prenatal screening was p < 0.001.

Prenatal screening for the presence of HBsAg was documented in 91.3% of women. In few women it was not clearly documented in the questionnaire whether test results were obtained prenatally or perinatally and those could not be included in this analysis of prenatal screening. Greek women were more likely to have had prenatal screening compare to non-Greek women Table (1).

Women being tested for the first time in the delivery room had higher prevalence of HBsAg(+) compare to those with documented prenatal care (4.2% versus 2.3%, p = 0.10) Table (2). Many of the women of Greek origin not tested prenatally however, reported past HBsAg(-) testing during a previous pregnancy. Although overall HBsAg carriage did not correlate with maternal age, we found that among women tested during prenatal care, seropositive women were slightly younger Table (3).

**Table 2:** HBsAg(+) prevalence by origin and time of 1^st ^documented screening for HBV.

Origin	Prenatal	Delivery room
Greek	34/2467 (1.4%)	3/174 (1.7%)
Albanian	20/227 (8.8%)	6/61 (9.8%)
Others*	11/135 (8.1%)	2/27 (7.4%)
Total	65/2829 (2.3%)**	11/262 (4.2%)**

*Others = non-Greek/non-Albanian women

** p = 0.10

**Table 3:** Maternal mean age in years (+/- SD) by HBsAg serostatus

	HBsAg (+)	HBsAg (-)	P value
Prenatal screening	28.69 (6.8)	29.5 (5.0)	P = 0.05
Delivery room	29.2 (6.3)	29.7 (4.8)	NS
Total	28.7 (6.6)	29.3 (5.1)	NS

One hundred twenty-two women (3.2%) were never tested (prenatally, perinatally, or during previous pregnancy). Most of them delivered in public clinics (77.6% versus 22.4% women, p < 0.001). Moreover not being ever tested for HBsAg correlated with maternal education; 16/72 (22.2%) of women never tested reported illiteracy compare to 50/2907 (1.7%) in the total population (p < 0.001). We found no association with ethnicity or maternal age. However, it is worth noticing that among those women not tested prenatally or perinatally, women of Greek origin were less likely to be primiparous compare to immigrant women (11,4% of Greek and 27,8% of immigrant women respectively p = 0.002). Finally, no geographic region was found to have a significantly higher prevalence of HBsAg or a lower percentage of screening pregnant women.

According to medical charts, in all identified cases of an HBsAg(+) mother, appropriate passive and active immunoprophylaxis was given within 24 hours. In one case a newborn of a HBsAg(-) woman was inadvertently given active and passive immunoprophylaxis.

## Discussion

Since selective screening of high risk pregnant women for HBsAg has failed to identify a significant proportion of HBV-infected mothers [2,18-20], prenatal HBsAg testing of all pregnant women has been recommended [2]. Universal prenatal testing would identify annually in the USA alone, an estimated 22,000 HBsAg (+) women and could prevent at least 6,000 chronic HBV infections in their offsprings [21].

The aim of the study was to evaluate the compliance of all Obstetric clinics with National Guidelines for the prevention of perinatal HBV infection with universal screening for HBsAg in pregnant women and the application of immunoprophylaxis in newborns to HBV carrier women. An excellent cooperation from all the obstetric clinics was observed. The number of deliveries during the study period, is in accordance with the annual birth rate reported (National Census 2001) as well as the number of Gurthrie cards completed over the study period and sent to the National Institute of Child Health. Furthermore, the demographics of women such as ethnicity percentages, correlated with those from our last National Census of 2001.

The overall prevalence of HBsAg (+) in pregnant women was 2.89%. Our result is in accordance with an earlier study in a Maternity Hospital in Athens [22]. The prevalence of HBsAg (+) was significantly lower among Greek women (1.7%) compare to non-Greek women. Especially high prevalence was found in women born in Albania (9.8%). As shown in previous epidemiologic studies in Greece, HBsAg-seropositivity is more prevalent in immigrant women compare to women of Greek origin [23,24]. Previously, Malamitsi-Puchner et al., found that immigrant women from Albania had HBsAg (+) prevalence of 13.4% [23] whereas another study in the early 1990's from Ioannina, a town in the border with Albania, examined the seroprevalence of HBV markers in refugees from Albania and showed an overall carrier rate of 22.2% with higher prevalence rates in younger immigrants [24]. In this study however, almost two thirds of the study population involved men. The authors suggest that low socioeconomic status and poor hygiene are important factors for horizontal transmission in this group. It is therefore possible that the lower prevalence found in our study compare to previous studies is due to the improvement of the socioeconomic status of immigrants. No geographical region showed significantly increased prevalence of HBsAg(+). Studies from other European countries show a decreased prevalence of chronic HBV-infection among pregnant women (1.0 –1.7%) compare to women delivering in Greece [25-27].

The overall incidence of prenatal screening was 91.3%, significantly higher that that documented in previous studies where only 63.1% had documentation of prenatal HBsAg testing and 10.3% had never had any prenatal examination [22]. In a recent Italian study, however, 91.8% of women had had prenatal screening for HBV-infection [25].

Greek women were more likely to have had prenatal screening for HBV infection Table (1). Women with no documented prenatal screening for HBsAg were more likely to be HBV chronically-infected (4.2% versus 2.3%, p = 0.10). This was true for Greek and Albanian women only. One could postulate that due to language barrier, the percentage of prenatal screening in non-Greek/non-Albanian women was underestimated since HBsAg-seropositivity was lower in this group of women tested for the first time in the delivery room Table (2).

As shown in a previous study [22], younger maternal age was an identifiable risk factor for HBsAg(+) in the subgroup of women with documented prenatal screening.

It is quite disturbing that during the two weeks of this study, while there was high awareness on HBV vertical transmission and therefore more women than usual might have been tested, 3.2% of women in our study had no documentation of prenatal or perinatal HBsAg screening. Risk factors identified included delivering in a Public Hospital and maternal illiteracy. Although deliveries were evenly distributed between public and private clinics (46.7% and 53.3% respectively), we found that more than 2/3 of women never tested delivered in a public hospital. Interestingly, in a recent Italian study women delivering in a Private Maternity Hospital were more likely not screened for HBV infection during pregnancy [25]. Maternal age, ethnicity and geographic region did not show any association with not ever being tested for HBsAg.

## Conclusion

Although the prevalence of HBsAg in Greek pregnant women is low and comparable to other European countries, immigrant women living in Greece which compose almost 20% of our childbearing population have significant higher prevalence rates. There has been significant improvement in the adherence to the antenatal screening guidelines over the past decade but, according to our results, there are still women who never get tested. Universal vaccination against HBV at birth and perinatal testing of all, not tested prenatally, women should be discussed with Public Authorities. The identification of HBsAg (+) mothers, even postnatally, is necessary for the administration of HBIG and most importantly for the appropriate follow up of these infants to ensure completion of vaccination against HBV and serologic testing according to guidelines.

## Abbreviations

Hepatitis B virus (HBV)

Hepatitis B Immune Globulin (HBIG)

Hepatitis B surface antigen (HBsAg)

## Competing interests

The author(s) declare that they have no competing interests.

## Authors' contributions

VP conceived of the study and participated in its design and coordination. CH participated in the design of the study and performed the statistical analysis. DK was responsible for the coordination of the study in Northern Greece. MT participated in the design and coordination of the study. All authors read and approved the final manuscript.

## Pre-publication history

The pre-publication history for this paper can be accessed here:


